# Intravenous dexmedetomidine pre-medication reduces the required minimum alveolar concentration of sevoflurane for smooth tracheal extubation in anesthetized children: a randomized clinical trial

**DOI:** 10.1186/s12871-018-0469-9

**Published:** 2018-01-17

**Authors:** Meiqin Di, Zhuqing Yang, Dansi Qi, Hongyan Lai, Junzheng Wu, Huacheng Liu, Xuefei Ye, Wangning ShangGuan, Qingquan Lian, Jun Li

**Affiliations:** 1 Department of Anesthesiology, The Second Affiliated Hospital and Yuying Children’s Hospital of WenZhou Medical University, No. 109 Xueyuan Western Road, Wenzhou, 325027 China; 2 Department of Anesthesiology, The Fourth Affiliated Hospital Zhejiang University School of Medicine, N1 Shangcheng Road, Yiwu, Zhejiang Province, People’s Republic of China; 3 Department of Pathology, The Second Affiliated Hospital and Yuying Children’s Hospital of WenZhou Medical University, 109 Xueyuan Western Road, Wenzhou, Zhejiang Province, People’s Republic of China; 4 Department of Anesthesia, Cincinnati Children’s Hospital Medical Center, Cincinnati, 3333 Burnet Ave, Cincinnati, OH 45229 USA

**Keywords:** Dexmedetomidine, Sevoflurane, Minimum alveolar concentration, Extubation, Pediatric

## Abstract

**Background:**

It has been known that Dexmedetomidine pre-medication enhances the effects of volatile anesthetics, reduces the need of sevoflurane, and facilitates smooth extubation in anesthetized children. This present study was designed to determine the effects of different doses of intravenous dexmedetomidine pre-medication on minimum alveolar concentration of sevoflurane for smooth tracheal extubation (MAC_EX_) in anesthetized children.

**Methods:**

A total of seventy-five pediatric patients, aged 3–7 years, ASA physical status I and II, and undergoing tonsillectomy were randomized to receive intravenous saline (Group D_0_), dexmedetomidine 1 μg∙kg^−1^ (Group D_1_), or dexmedetomidine 2 μg∙kg^−1^ (Group D_2_) approximately 10 min before anesthesia start. Sevoflurane was used for anesthesia induction and anesthesia maintenance. At the end of surgery, the initial concentration of sevoflurane for smooth tracheal extubation was determined according to the modified Dixon’s “up-and-down” method. The starting sevoflurane for the first patient was 1.5% in Group D_0_, 1.0% in Group D_1_, and 0.8% in Group D_2_, with subsequent 0.1% up or down in next patient based on whether smooth extubation had been achieved or not in current patient. The endotreacheal tube was removed after the predetermined concentration had been maintained constant for ten minutes. All responses (“smooth” or “not smooth”) to tracheal extubation and respiratory complications were assessed.

**Results:**

MAC_EX_ values of sevoflurane in Group D_2_ (0.51 ± 0.13%) was significantly lower than in Group D_1_ (0.83 ± 0.10%; *P* < 0.001), the latter being significantly lower than in Group D_0_ (1.40 ± 0.12%; *P* < 0.001). EC_95_ values of sevoflurane were 0.83%, 1.07%, and 1.73% in Group D_2_, Group D_1_, and Group D_0_, respectively. No patient in the current study had laryngospasm.

**Conclusion:**

Dexmedetomidine decreased the required MAC_EX_ values of sevoflurane to achieve smooth extubation in a dose-dependent manner. Intravenous dexmedetomidine 1 μg∙kg^−1^ and 2 μg∙kg^−1^ pre-medication decreased MAC_EX_ by 41% and 64%, respectively.

**Trial registration:**

Chinese Clinical Trial Registry (ChiCTR): ChiCTR-IOD-17011601, date of registration: 09 Jun 2017, retrospectively registered.

## Background

Dexmedetomidine, a highly selective α_2_-adrenergic agonist is an effective pre-medication [1, 2] to deepen the level of anesthesia and consequently reduce the requirements of anesthetics in children [3, 4]. In our previously study [5], it has been proveded that a single dose of intravenous dexmedetomidine given before induction either at 1 μg∙kg^−1^ or 2 μg∙kg^−1^ facilitated deep extubation in the presence of low concentrations of inhaled sevoflurane.

Smooth tracheal extubation can be performed in pediatric patients who are anesthetized with high concentrations of sevoflurane [6]. The depth of sevoflurane anesthesia determines the optimal timing and quality of tracheal extubation. Endotracheal tube removal in a patient anesthetized at a level of deeper than optimal carries the high risks of persistent suppression of pharyngeal reflexes and the unprotected airway, which may cause airway obstruction and pulmonary aspiration. However, if the anesthesia is lighter than optimal, the upper airway reflexes may become irritated during extubation, and as a consequence, some life-threatening complications could occur, such as apnea, coughing, laryngospasm, or bronchospasm. It is therefore important that the endotracheal tube should be removed while the patient is still anesthetized under the least amount of anesthesia on board, definitely with the minimal untoward effects.

Now, there is no study which has been performed to quantitate the minimum alveolar concentration of sevoflurane for smooth extubation (MAC_EX_) in patients pre-medicated with dexmedetomidine. We conducted this study to determine the effects of two different dosages of intravenous dexmedetomidine as pre-medication on optimal MAC_EX_ of sevoflurane using a modified Dixon’s up-and-down method.

## Methods

The study protocol was approved by the Hospital Ethics Committee of the Second Affiliated Hospital and Yuying Children’s Hospital of WenZhou Medical University (Ethical Committee CI 56 /2017). A total of 75 children, ASA physical status I or II, aged 3–7 years old, scheduled to undergo tonsillectomy during the period of April 2017 to June 2017, were enrolled in this observational study (trial registry identifier, ChiCTR-IOD-17011601) after informed consent was obtained from parents or guardians. Exclusion criteria included suspected difficult airway, asthma, ongoing upper respiratory infection, or other congenital or neurological disease.

Using a computer-generated random number sequences (SPSS 21.0, SPSS Inc., U.S.), patients were randomly allocated to one of the three groups: saline infusion (Group D_0_), dexmedetomidine 1 μg∙kg^−1^ infusion (Group D_1_) or dexmedetomidine 2 μg∙kg^−1^ infusion (Group D_2_). The allocation ratio was 1:1. An anesthesia nurse prepared the drug or saline solution, but did not participate in following study protocol. An attending anesthesiologist performed the anesthesia and another anesthesiologist, worked as research observer, to watch the process of extubation and collected study parameters.

All patients were instructed to follow the ASA preoperative fasting guideline and patient arrived at the pre-anesthesia holding room in the presence of one parent approximately 20 min before anesthesia. The local anesthetic cream was applied and an intravenous (IV) line was placed. The noninvasive arterial blood pressure, electrocardiogram, oxygen saturation (SPO_2_), heart rate (HR), and respiratory rate (RR) were measured initially as baseline and continued to be monitored throughout the whole study. Patients received saline or dexmedetomidine infusion over 10 min in pre-op area according to their group assignments. And then, the patients were transferred to the operating room. General anesthesia was induced via a face mask with inhalation of 8% sevoflurane in oxygen at 5 L∙min^−1^. The tracheal intubation was performed without using any IV muscle relaxants when the Anesthesia Stage III had been confirmed (conjugated and small pupils and floppy abdominal muscles). The Adequate depth of anesthesia was maintained with the adjustment of sevoflurane, and patients were kept breathing spontaneously throughout the surgery. The inspired/end-tidal sevoflurane and carbon dioxide concentrations were measured with a gas monitor (Dräger, Lübeck, Germany). Adequate spontaneous ventilation was defined as a normal end-tidal carbon dioxide partial pressure (ETCO_2_) waveform and an ETCO_2_ concentration less than 6.0 kPa [7]. The manually assisted ventilation was provided when the ETCO_2_ concentration was over 7.2 kPa [8].

Before surgical incision, fentanyl (0.5 μg∙kg^−1^), ondansetron hydrochloride (0.1 mg∙kg^−1^), and dexamethasone (0.2 mg∙kg^−1^) were given intravenously to all children. At the end, 0.2% Ropivacaine (0.25 mg/kg) with epinephrine (1:200,000) was infiltrated to the tonsil bed to provide additional postoperative analgesia. After surgery, patient was turned on his or her side, and the oropharynx was gently suctioned. The patient was breathing at a predetermined concentration of sevoflurane before extubation for at least 10 min to establish an equilibrium between the alveoli and brain tissue. Here, it was set to 1.5%, 1.0%, and 0.8% for the first patient in groups D_0_, D_1_, and D_2_, respectively [5]. Once a regular spontaneous respiratory pattern had been confirmed by capnography waveforms, the tracheal tube cuff was deflated and the endotracheal tube was gently removed at a speed of 1 cm s^−1^ by a pediatric attending anesthesiologist. Sevoflurane was discontinued and oxygen (8 L∙min^−1^) was administered over facemask immediately after extubation. An oral airway was placed only if the patient showed signs of obstructed airway. In addition, propofol 2 mg∙kg^−1^ and continuous positive airway pressure (CPAP) was used if patients developed breath holding or laryngospasm.

The concentration of sevoflurane for tracheal extubation was determined according to the modified Dixon’s up-and-down approach, which is described as follows [9]. Smooth tracheal extubation was defined as no gross purposeful muscular movement, such as coughing, within 1 min of tracheal tube removal [10]. Extubation was considered non-smooth if the patient showed coughing, breath holding, or laryngospasm immediately after extubation. If smooth extubation was achieved in a previous patient at a predetermined concentration of sevoflurane, the next patient would receive 0.1% lower of sevoflurane during extubation; if the extubation was not smooth, the next patient would be given 0.1% higher of sevoflurane. This process of 0.1% stepwise increments or reduction was repeated until all groups had accumulated six independent turning points of consecutive subjects in which non-smooth tracheal extubation conditions were followed by smooth extubation. The patients with different responses, either “smooth” or “not smooth” to tracheal extubation were constituted a crossover. Each patient was only involved in one single crossover. A research observer was assigned to evaluate the responses of extubation and record any airway events (coughing, breathe holding, laryngospasm, hypoxemia, and airway obstruction). Duration of anesthesia (from sevoflurane induction to sevoflurane discontinuation), duration of surgery, and the time from the start of dexmedetomidine or saline infusion to extubation were recorded.

When the airway and adequate spontaneous ventilation were assured after extubation, patients were transferred to the post-anesthesia care unit (PACU) and kept in the PACU until they were calm and free of pain or nausea assessed by an Aldrete score of 9–10 [11].

## Statistical analysis

The size of sample was calculated based on the MAC_EX_ of sevoflurane in children, which is approximately 1.5% [5, 6]. Twenty-five patients were required for each group in order to determine that pre-medicine with dexmedetomidine would reduce MAC of sevoflurane for smooth extubation between the groups with 80% power and a type I error of 5%. The enrollment of subjects were stopped when each group had accumulated six independent turning points of consecutive subjects as described in the modified Dixon’s ‘up-and-down’approach [9].

The SPSS 21.0 for Windows (SPSS Inc., U.S.) was used to perform the statistical analyses. MAC_EX_ was obtained by calculating the mean of midpoint concentration of all crossovers in each group. Standard deviation (SD) of MAC_EX_ was the SD of the crossover midpoint in each group. The success of the smooth tracheal extubation was analyzed using the following logistic regression model to determine sevoflurane end tidal concentrations where 50% (EC_50_ = MAC) and 95% (EC_95_) were successful.

*P* (probability of smooth extubation) is here expressed using the following formula: P = e^B0 + B1X^/1 + e^B0 + B1X^, where X = end-tidal sevoflurane concentration, B0 = regression intercept constant, and B1 = coefficient for sevoflurane. To determine MAC_EX_ (EC_50_) or EC_95_ values for a smooth extubation, the probability of ‘smooth’ was evaluated at *P* = 0.5 or *P* = 0.95, and the equation was solved for X.

The results are here expressed in terms of [mean ± standard deviation (SD)]. Parametric data among groups were analyzed using one-way analysis of variance and Mann–Whitney rank-sum test, depending on the distribution of the data. Nominal data were analyzed using either χ2 or Fisher’s exact tests. Differences at *P* < 0.05 were considered statistically significant.

## Results

**Fig. 1:**
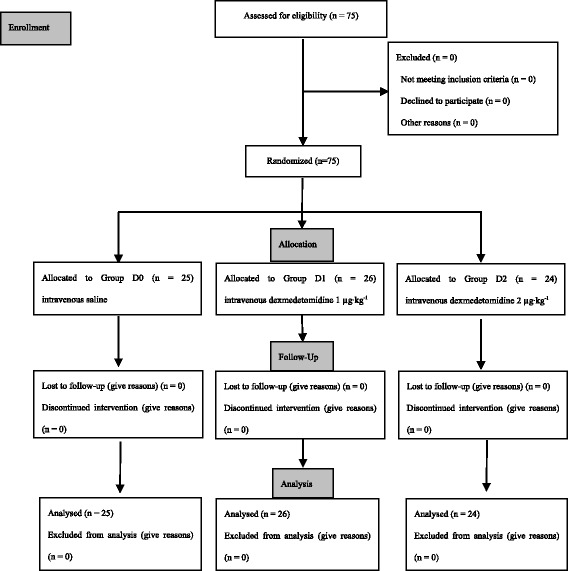
CONSORT flow diagram

A total of 75 eligible children were enrolled into the study. Consolidated Standards of Reporting Trials (CONSORT) flow diagram is shown in Fig. 1. All tracheal intubation procedures were performed successfully, and no patient was excluded from the statistical analyses. There were no dropouts or protocol violations, and complete datasets were available for all children. The demographic data (age, sex, and weight), the time from the start of pre-medication to extubation, and the duration of surgery and anesthesia were similar among three groups (*P* > 0.05; Table 1).

**Table 1:** Patient data and clinical characteristics

Variable	Group D_0_ (*n* = 25)	Group D_1_ (*n* = 26)	Group D_2_ (*n* = 24)
Age (y)	5.3 ± 1.3	5.0 ± 1.1	5.1 ± 1.0
Sex (male/female)	12/13	13/13	13/11
Weight (kg)	19.5 ± 3.4	19.6 ± 3.7	19.8 ± 3.4
Duration of surgery (min)	18.5 ± 3.4	19.3 ± 3.4	18.6 ± 4.0
Duration of anesthesia (min)	32.8 ± 3.7	34.4 ± 4.0	33.9 ± 4.1
Time from initiation of dexmedetomidine/saline infusion to tracheal removal (min)	47.0 ± 3.6	46.6 ± 4.0	47.2 ± 4.5

Data are expressed as mean ± standard deviation (SD) (One-way ANOVA test) or number of patients (Chi Square test)

Group D_0_ = saline group, Group D_1_ = dexmedetomidine group (1 μg·kg^−1^), Group D_2_ = dexmedetomidine group (2 μg·kg^−1^)

**Fig. 2:**
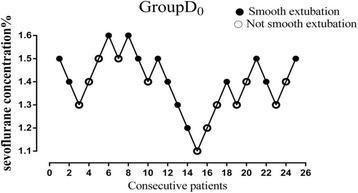
Responses of 25 consecutive saline premedicated children in Group D_0_. (●) indicates smooth tracheal extubation and (○) indicates non-smooth tracheal extubation

**Fig. 3:**
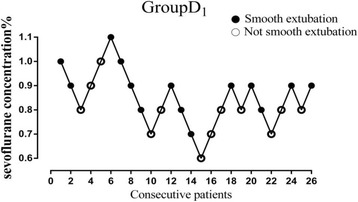
Responses of 26 consecutive dexmedetomidine 1 μg·kg^−1^ premedicated children in Group D_1_. (●) indicates smooth tracheal extubation and (○) indicates not smooth tracheal extubation

**Fig. 4:**
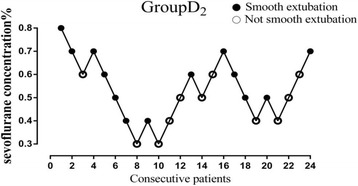
Responses of 24 consecutive dexmedetomidine 2 μg·kg^−1^ premedicated children in Group D_2_. (●) indicates smooth tracheal extubation and (○) indicates non-smooth tracheal extubation

**Fig. 5:**
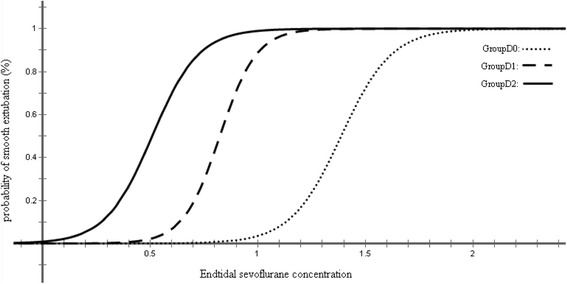
Dose-response curves for sevoflurane plotted of individual end-tidal concentrations and reactions to smooth tracheal extubation in groups D_0,_ D_1_, and D_2_ (from logistic analyses)

Figures 2, 3, and 4 showed individual data for Group D_0_, Group D_1_, and Group D_2_, which were obtained using the up-and-down method. There were total nine, ten and eight crossovers in Group D_0_, Group D_1_, and Group D_2_, respectively. The MAC_EX_ values of sevoflurane in Group D_2_ (0.51 ± 0.13%) were significantly lower than in Group D_1_ (0.83 ± 0.10%; *P* < 0.001), the latter being significantly lower than in Group D_0_ (1.40 ± 0.12%; *P* < 0.001). Logistic regression curves of the probability of smooth tracheal extubation were shown in Fig. 5. According to logistic regression curves, MAC_EX_ (EC_50_) values of sevoflurane for smooth extubation were 1.39%, 0.82%, and 0.51% in Group D_0_, Group D_1_, and Group D_2_, respectively. Sevoflurane EC_95_ values for smooth extubation were 1.73%, 1.07%, and 0.83% in these three groups, respectively.

The most common event for non-smooth extubation was coughing. After extubation, two patients in Group D_0_ experienced transient breathe holding with a transient reduction in SpO_2_, and then, SpO_2_ returned to 98% and above within 1 min after treated with CPAP. Three patients in Group D_0_, one patient in Group D_1_, and two patients in Group D_2_ experienced airway obstruction and required oral airway placement. No patient had developed laryngospasm or was re-intubated in all three groups.

Mean arterial pressure and HR data are reported in Table 2. Both MAP and HR in the groups D_1_ and D_2_ were lower than its baselines after dexmedetomidine infusion and before extubation (*P* < 0.05). They were also lower than those in Group D_0_ (*P* < 0.05). Both MAP and HR in Group D_0_ were significantly higher at 1 min and 5 min after extubation than just before extubation (*P* < 0.05). They were also higher than in Groups D_1_ and D_2_ (*P* < 0.05).

**Table 2:** Hemodynamic changes

	MAP (mmHg)			*P* value	HR (b/min)			*P* value
	GroupD_0_ (*n* = 25)	Group D_1_ (*n* = 26)	Group D_2_ (*n* = 24)		Group D_0_ (*n* = 25)	Group D_1_ (*n* = 26)	Group D_2_ (*n* = 24)	
Baseline	81 ± 7	80 ± 6	79 ± 6	0.728	108 ± 13	109 ± 12	107 ± 12	0.924
Just after pre-medicine	81 ± 7	72 ± 8*^	72 ± 6* ^	<0.000	109 ± 13	94 ± 10*^	92 ± 9*^	<0.000
Just before extubation	78 ± 7	74 ± 8*^	73 ± 7*^	<0.02	105 ± 10	99 ± 10*^	96 ± 9*^	<0.009
One min after extubation	84 ± 5^*#*^	76 ± 7^	76 ± 7^	<0.002	115 ± 13^*#*^	104 ± 9^	102 ± 9^	<0.006
Five min after extubation	82 ± 7^*#*^	76 ± 7^	76 ± 7^	<0.029	115 ± 13^*#*^	104 ± 10^	101 ± 10^	<0.008

Data are expressed as mean ± standard deviation (SD) (One-way ANOVA test)

*MAP* = arterial blood pressure, *HR* = heart rate

Group D_0_ = saline group, Group D_1_ = dexmedetomidine group (1 μg·kg^−1^), Group D_2_ = dexmedetomidine group (2 μg·kg^−1^)

*P < 0.05 compared with values of baseline, ^*#*^*P < 0.05* compared with values just before extubation, *^ P < 0.05* compared with Group D_0_

## Discussion

Our study demonstrated that a single intravenous pre-medication of dexmedetomidine was associated with dose-dependent reduction in the MAC_EX_ of sevoflurane in anesthetized children undergoing tonsillectomy. Intravenous dexmedetomidine 1 μg∙kg^−1^ and 2 μg∙kg^−1^ pre-medication reduced MAC_EX_ by 41% and 64%, respectively.

Adequate suppression of airway reflexes necessary for facilitating safe and smooth extubation can be fulfilled with a few of pharmacological interventions including dexmedetomidine, remifentanil [12] and clonidine [13]. However, remifentanil is less ideal with its risks of respiratory depression and very short analgesic and sedative effects. Both Dexmedetomidine and Clonidine mainly are α_2_ adrenergic receptor agonists with weak action on α_1_ receptors. But, the ratio of selectivity to α_2_ receptors for Dexmedetomidine is eight to ten times greater than that of clonidine [14] leading to better sedative effect and less side effect of dexmedetomidine.

Dexmedetomidine has sedative and analgesic properties and it is a safe and effective drug used in many clinical scenarios because it does not cause respiratory depression and won’t significantly affect hemodynamics [12]. Dexmedetomidine has been shown to increase tolerance to the presence of endotracheal tubes and diminish airway responses to laryngeal and tracheal irritation during endotracheal tube removal [15, 16]. Some clinical studies have reported that dexmedetomidine could deepen the effect of volatile anesthesia, produce a dose-dependent decrease in the minimum alveolar concentration and reduce the MAC of sevoflurane in children during laryngeal mask airway insertion [3], laryngeal mask airway removal [17], and endotracheal intubation [18]. The reduction of dexmedetomidine-induced sevoflurane MACs varies from 20% to 60% [3, 17, 18]. The aim of our study was to investigate the dose-related effects of intravenous dexmedetomidine as pre-medication on the MAC of sevoflurane for smooth endotracheal extubation. We found that a single intravenous injection of dexmedetomidine (1 μg∙kg^−1^ or 2 μg∙kg^−1^) as pre-medication could significantly reduce the MAC_EX_ values of sevoflurane in children by 41% and 64% in Groups D_1_ and D_2_, respectively, relative to the saline group. The minimum alveolar concentrations of sevoflurane for smooth tracheal extubation were 0.82% in Group D_1_ and 0.51% in Group D_2_, which met the criteria for smooth extubation closely. The EC_95_ values of sevoflurane, 1.07% in Groups D_1_ and 0.83% in Group D_2,_ for smooth tracheal extubation indicate that tracheal extubation should not be attempted until the sevoflurane concentration has reached at least 1.07% and 0.83%. The higher the dexmedetomidine dose administered, the lower the concentration of sevoflurane needed for smooth extubation.

Inomata et al. reported that the MAC_EX_ value of sevoflurane in children was 1.64% with the administration of N_2_O but without systematic analgesics [14]. In this present study, we found the MAC_EX_ value of sevoflurane for smooth extubation to be 1.40% in Group D_0_. The difference in MAC requirement of sevoflurane between the two studies might be attributable to the use of fentanyl and local anesthetic blocks in our study. Potent analgesic drugs [19] and regional blocks [20] have been shown to reduce the requirement of inhaled anesthetic agents.

Intravenous administration of dexmedetomidine may have some effects on hemodynamics. In the present study, we observed relatively slow HR and lower blood pressure just after pre-medicine and before extubation in dexmedetomidine group, but the deviation did not exceed 20% of the baseline values. Immediately after extubation, HR and MAP in Groups D_1_ and D_2_ increased only slightly, which indicated that the stress response to the maneuver of extubation was suppressed by dexmedetomidine. By contrast, significant increases of those hemodynamic parameters were observed in Group D_0_ during extubation. The minor changes of hemodynamics in dexmedetomidine groups during extubation were highly beneficial to patients undergoing tonsillectomy and other surgical procedures.

The minimum alveolar concentration is influenced by age and arterial CO_2_ tension [21, 22], and MAC values of sevoflurane remain constant in children aged 6 months to 12 years [22]. For this reason, we maintained normal ETCO_2_ concentration throughout sevoflurane anesthesia in the current study.

The study has its limitations that should be considered. First, this is a single-center study with small sample size, which may affect the strength of the conclusions. More studies are warranted to further substantiate our evidence. Second, out study protocol required an additional 10 min of anesthesia beyond the 20 min surgery to allow sevoflurane fully equilibrated between brain and alveoli before extubation. This intentionally prolonged anesthesia may not be applicable to other extubation settings. Third, the IV fentanyl and the local anesthetic block to the tonsillar beds had been integrated into the general anesthesia in our study. The variation of opioid dosage and the preference of regional block by individual anesthetists could change the optimal MAC_EX_ of sevoflurane. Fourth, the differences in the skills and training methods of anesthesiologists may also affect the quality of extubation and the incidence of perioperative respiratory complications [23]. In our study, all tracheal extubation procedures were performed by an attending pediatric anesthetist with fifteen years of working experience in order to minimize operational discrepancies.

## Conclusions

In summary, intravenous dexmedetomidine pre-medication produced a dose-dependent decrease in the minimum alveolar concentration of sevoflurane for smooth tracheal extubation of in children.

## Abbreviations

ASA: American Society of Anesthesiologists
CPAP: continuous positive airway pressure
ETCO2: End-tidal carbon dioxide
MACEX: Minimum alveolar concentration of sevoflurane for smooth tracheal extubation
PACU: Post-anesthesia care unit
SPO2: oxygen saturation
